# Pathogenic Inflammation and Its Therapeutic Targeting in Systemic Lupus Erythematosus

**DOI:** 10.3389/fimmu.2015.00550

**Published:** 2015-10-28

**Authors:** Timothy A. Gottschalk, Evelyn Tsantikos, Margaret L. Hibbs

**Affiliations:** ^1^Leukocyte Signalling Laboratory, Department of Immunology and Pathology, Alfred Medical Research and Education Precinct, Monash University, Melbourne, VIC, Australia

**Keywords:** inflammation, SLE/lupus, nephritis, immunopathology, interleukin-6, proinflammatory cytokines, lupus models, therapeutics

## Abstract

Systemic lupus erythematosus (SLE, lupus) is a highly complex and heterogeneous autoimmune disease that most often afflicts women in their child-bearing years. It is characterized by circulating self-reactive antibodies that deposit in tissues, including skin, kidneys, and brain, and the ensuing inflammatory response can lead to irreparable tissue damage. Over many years, clinical trials in SLE have focused on agents that control B- and T-lymphocyte activation, and, with the single exception of an agent known as belimumab which targets the B-cell survival factor BAFF, they have been disappointing. At present, standard therapy for SLE with mild disease is the agent hydroxychloroquine. During disease flares, steroids are often used, while the more severe manifestations with major organ involvement warrant potent, broad-spectrum immunosuppression with cyclophosphamide or mycophenolate. Current treatments have severe and dose-limiting toxicities and thus a more specific therapy targeting a causative factor or signaling pathway would be greatly beneficial in SLE treatment. Moreover, the ability to control inflammation alongside B-cell activation may be a superior approach for disease control. There has been a recent focus on the innate immune system and associated inflammation, which has uncovered key players in driving the pathogenesis of SLE. Delineating some of these intricate inflammatory mechanisms has been possible with studies using spontaneous mouse mutants and genetically engineered mice. These strains, to varying degrees, exhibit hallmarks of the human disease and therefore have been utilized to model human SLE and to test new drugs. Developing a better understanding of the initiation and perpetuation of disease in SLE may uncover suitable novel targets for therapeutic intervention. Here, we discuss the involvement of inflammation in SLE disease pathogenesis, with a focus on several key proinflammatory cytokines and myeloid growth factors, and review the known outcomes or the potential for targeting these factors in SLE.

## Introduction

**Figure 1:**
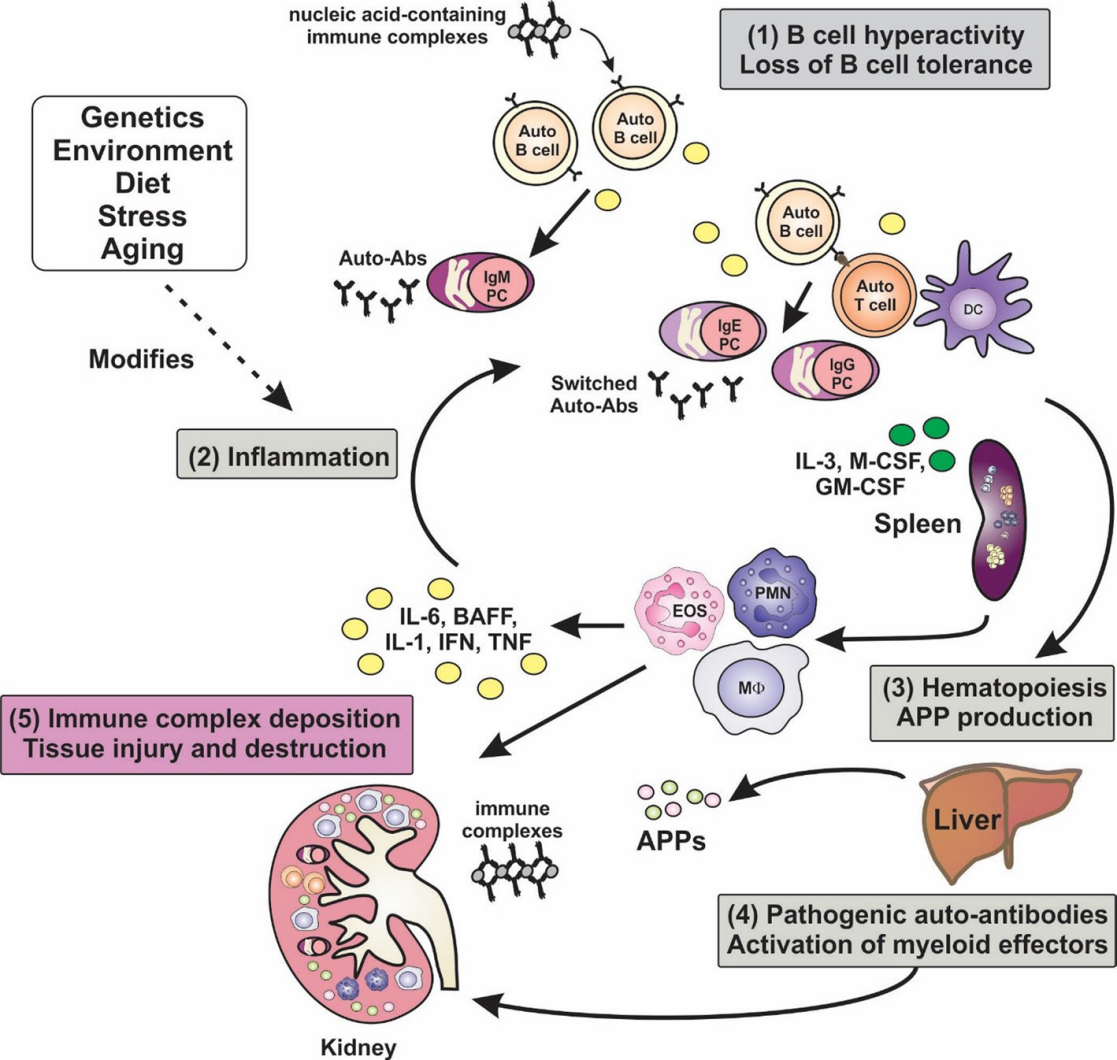
**Inflammation is a key factor in the pathogenesis of lupus**. A hallmark of lupus is the presence of hyperactive B cells and loss of B-cell tolerance. Immune complexes containing nucleic acid autoantigens can engage and activate endosomal TLRs and promote inflammation in SLE. Plasma cell expansion and the production of autoantibodies are also features, although the autoantibodies are benign unless generated in an inflammatory milieu, wherein class-switching to pathogenic isotypes occurs. Proinflammatory cytokines not only drive T-cell activation and dendritic cell maturation, but they can stimulate extramedullary hematopoiesis leading to expansion of innate immune cells, and they can induce the production of acute-phase proteins (APPs). Autoantibodies become deposited in tissues such as the glomeruli of the kidney, leading to the activation of myeloid effector cells via Fcγ and complement receptors, leading to tissue destruction. Numerous factors, including genetic make-up, environment, diet, and stress, can modify disease course and severity.

Systemic lupus erythematosus (SLE, lupus) is a B-cell-mediated autoimmune disease characterized by the generation of autoantibodies against nuclear antigens and a type III hypersensitivity reaction leading to chronic systemic inflammation. The disease is polygenic and highly complex, requiring interplay between multiple immunopathogenic factors including host autoantigens and both cellular and humoral immune components that contribute to the generation of a hyperinflammatory environment resulting in organ and tissue damage (Figure 1). Deposition of circulating autoantibody–autoantigen complexes can occur in various tissues and organs of the body, resulting in a local inflammatory response and severe tissue destruction. Sites often affected include skin (cutaneous lupus), the nervous system (CNS lupus), joints and muscles (rheumatoid lupus, rhupus), and the kidney (renal lupus, lupus nephritis), which contributes most significantly to disease morbidity (1). Disease progression is non-linear and follows a relapse-remitting course, and due to its heterogeneous nature, it can vary widely from patient to patient, making diagnosis and treatment a challenge (1). The current diagnostic criteria require a patient to present with 4 out of 11 symptoms/disorders, including cutaneous rashes, inflammation of the pleura or pericardium, inflammation of joints and muscles, renal and/or neurologic disorders, hematologic and immunologic disorders, and most significantly, autoantibodies specifically targeting nuclear antigens (such as double-stranded DNA, small nuclear riboproteins, chromatin, and histone proteins), or to a lesser extent, cytoplasmic antigens (2). SLE has a strong genetic component with a high familial concordance, but environmental triggers such as UV radiation, stress, medication, or infection can contribute to disease onset. Genetic analyses, and more recently, genome-wide association studies, have uncovered various human SLE susceptibility genes that are normally responsible for maintaining immune system tolerance and homeostatic processes. These include antigen processing and presentation (*HLA*, *TAP1/2*), clearance of apoptotic debris (*C1q*, *Dnase1*), leukocyte cell surface receptors (*FCGRI/II/III*, *ITGAM*), and cell signaling and gene transcription molecules (*LYN*, *BLK*, *PTPN22*, *STAT4*, *IRF5*) (3, 4). SLE affects approximately one in every 2,500 individuals, although this can be highly variable based on geographical location, ethnicity, and sex [reviewed in Ref. (5, 6)]; SLE is most commonly seen in females (9:1 prevalence) with disease onset typically around child-bearing age and in those of non-Caucasian ethnicity.

As SLE is highly complex, multifactorial, and manifests in an array of pathologies, the disease has been difficult to study in humans. Mouse models that mimic aspects of SLE pathology and pathogenesis include the naturally occurring mutants MRL^lpr/lpr^, NZB/W F_1_, congenic BSXB/Yaa, NZM2410, B6.sle1.sle2.sle3, B6.sle1.Yaa, transgenic (Tg) overexpression of BAFF in BAFF-Tg, and induced models such as pristane and ALD-DNA [reviewed in Ref. (7, 8)]. One well-studied model of SLE is the Lyn-deficient mouse (Lyn^−/−^) (9), which exhibits clinical, pathological, and biochemical features seen in human SLE [comprehensively reviewed in Ref. (10)]. Lyn^−/−^ mice develop serum antinuclear antibodies (ANAs) and acquire glomerulonephritis due to immune complex deposition in kidney (9), which leads to renal dysfunction (11). The mice show hematologic disorders such as lymphopenia and thrombocytopenia (9) and develop splenomegaly and lymphadenopathy (12). Similar to SLE patients, Lyn^−/−^ mice also have hyperactive B lymphocytes and altered signaling pathways (13–15). *Lyn* is a haploinsufficiency gene in autoimmunity (16), and it is implicated in human disease (10). Much of our current understanding of SLE disease pathogenesis and many preliminary therapeutic studies for SLE have come from the identification, analysis, or testing of these mouse models [reviewed in Ref. (17, 18)].

### Inflammation and Immunopathology of Lupus Nephritis

**Figure 2:**
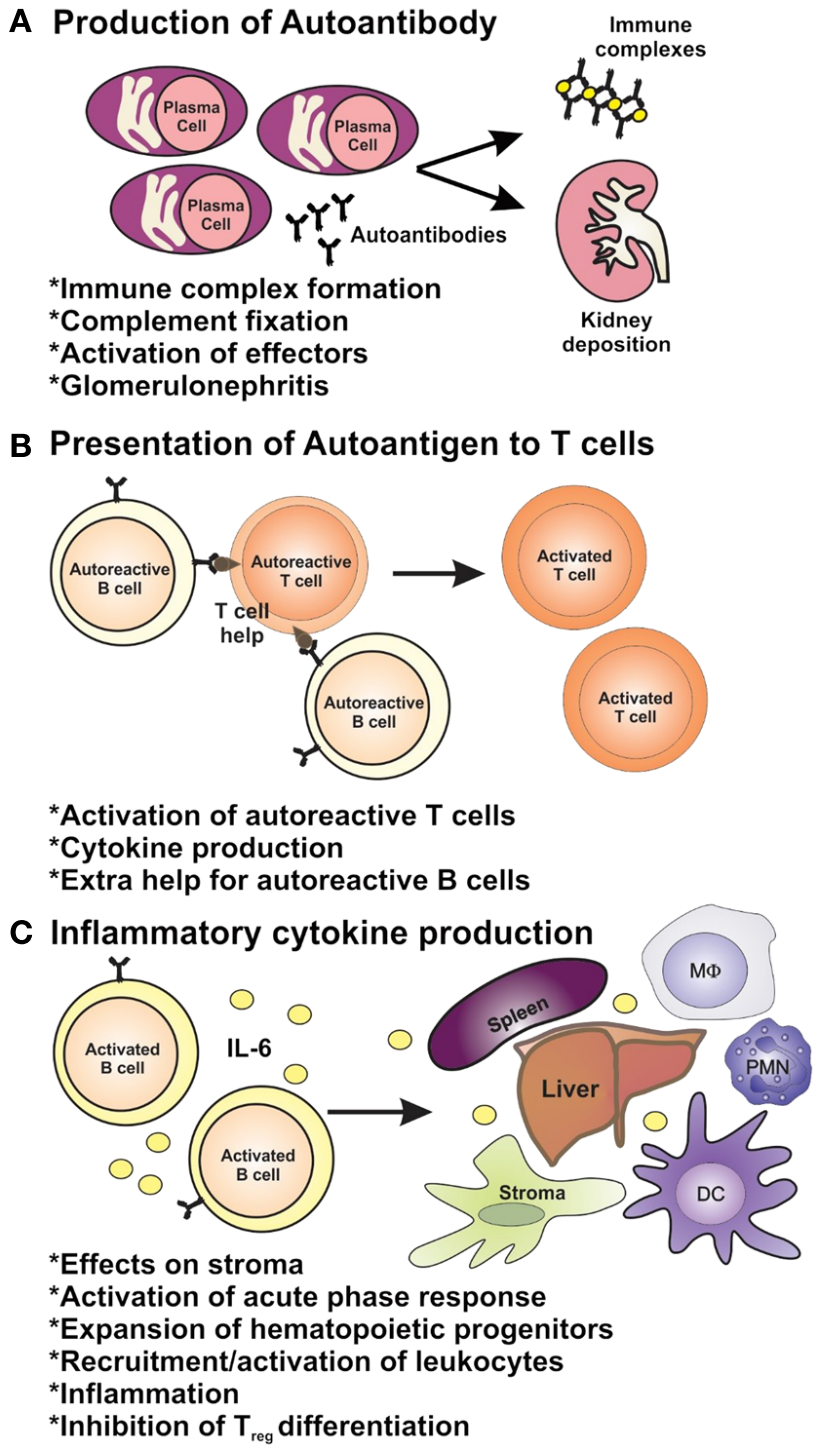
**The roles of B cells in lupus pathogenesis**. B cells have multiple roles in autoimmunity through **(A)** their ability to produce autoantibodies and **(B)** via their role as antigen-presenting cells and **(C)** as producers of inflammatory cytokines.

One or more mechanisms of B-cell tolerance are lost in SLE, allowing for the production of ANAs by plasma cells [reviewed in Ref. (19, 20)] (Figure 1). Upward of 90% of SLE patients have elevated titers of serum ANAs, on average 2–3 years prior to clinical onset of SLE (21), with 30–70% of SLE patients developing life-limiting renal disease (22). The temporal delay between autoantibody development and disease onset coupled with incomplete penetrance of ANA-mediated disease suggests that pathogenesis of autoantibody-driven nephritis is conditional upon other factors, such as antigen availability, a pre-established inflammatory environment, and T-cell-mediated antibody isotype switching (**Figure 1**). While a hallmark of inflammation is the elevation in levels of C-reactive protein (CRP), many lupus patients demonstrate normal or even reduced levels of CRP. CRP is involved in the clearance of apoptotic cells [reviewed in Ref. (23, 24)], and if they are inadequately cleared, this can expose nuclear antigens allowing for ANAs to extensively bind and form immune complexes (ICs). Such ICs can deposit in the basement membrane of the glomerular microvessels (25), resulting in activation of the alternative complement pathway and recruitment of proinflammatory macrophages and dendritic cells to the glomeruli via chemotactic signaling which upregulate inflammatory cytokine production and activate autoreactive T-cell subsets through antigen presentation and costimulation (Figure 1) (22, 26). Endosomal toll-like receptors (TLR)-7 and TLR-9 in activated B cells, plasmacytoid dendritic cells, and macrophages can respond to internalized self ICs containing nucleic acids, which can contribute to the initiation and perpetuation of the inflammatory cascade (Figure 1) [reviewed in Ref. (27)]. CD4^+^ T helper cells play several key roles in the pathogenesis of lupus nephritis: T helper 1 (T_h_1) cells are responsible for high-level production of proinflammatory cytokines, such as interferon-γ (IFN-γ), which stimulates dendritic cell and myeloid cell production of interleukin-(IL)-1, IL-6, IL-12, IL-18, TNF-α, and BAFF creating a perpetual proinflammatory loop; T helper 2 cells (T_h_2) produce cytokines (IL-4, IL-5), which induce antibody isotype class-switching leading to the production of high affinity, pathogenic autoantibodies [reviewed in Ref. (28, 29)]; T_h_17 cells also provide B-cell support, promote plasma cell differentiation and pathogenic autoantibody production and myeloid cell hyper-activation which drives systemic inflammation (30, 31); T follicular helper cells (T_FH_) are now also known to contribute to autoimmune germinal center reactions or autoantibody production in lupus-prone mice and SLE patients (32, 33) [reviewed in Ref. (34)]. Aside from autoantibody production (Figure 2A), autoreactive B cells contribute to the pathogenesis of lupus nephritis via two supportive mechanisms: B cells can activate autoreactive T cells through antigen presentation and costimulation (Figure 2B) and they can produce cytokines including IL-6, a proinflammatory cytokine able to drive inflammation and inhibit the generation of autoimmune suppressive regulatory T cells (Treg) (Figure 2C) (22, 29). As well as T-cell-induced antibody isotype switching within germinal centers, evidence shows ectopic germinal center-like congregations within the glomeruli of SLE patients suggesting B cells may undergo local somatic hypermutation of immunoglobulin (Ig) variable region genes generating both higher affinity autoantibodies and memory B cells (35). Inflammation and cytotoxicity caused by the immune response generated against glomerular ICs results in progressive renal tissue damage including immune cellular influx and progressive fibrotic, sclerotic, and necrotic lesions (36). As a consequence of this, patients suffer glomerular degeneration and reduced kidney function, which may result in end-stage renal failure requiring dialysis or transplantation (37). Currently, there is an incomplete understanding of the factors driving pathogenesis in lupus nephritis, which hinders the development of novel, targeted therapeutics.

### Current Treatments and Lack of Clinical Success of B-Cell-Directed Therapies

Treatment of SLE has remained fairly consistent over the past 50 years utilizing non-specific anti-inflammatory agents such as non-steroidal anti-inflammatory drugs and the immune cell modulating hydroxychloroquine for mild disease, and broad-spectrum immunosuppressants/anti-inflammatories such as corticosteroids, azathioprine, cyclophosphamide, or mycophenolate during flares or severe disease with organ involvement (38). These therapies can have severe, dose-limiting toxicities and undesirable side effects, and some patients lack an adequate response, highlighting the need for a treatment regime tailored to individual patients (38). Development and trial of novel targeted treatments for SLE have been difficult due to the complexity and limited knowledge of disease etiology, loose diagnostic criteria, various disease manifestations, and the diverse heterogeneity of patients [reviewed in Ref. (39)].

Despite promising results in animal models (40), therapeutic targeting of B cells in SLE has had minimal success, especially in patients with lupus nephritis. B-cell depletion using rituximab, a monoclonal antibody targeting the B-cell-specific receptor CD20 (anti-CD20 mAb), was unsuccessful at meeting primary and secondary endpoints in stage II/III SLE clinical trials despite showing initial promising results (41, 42). Ocrelizumab, a humanized anti-CD20 mAb, also failed in phase III clinical trials demonstrating insignificant renal improvement and increases in severe infection of patients (43). Inhibition of B-cell survival pathways by neutralizing the survival receptor TACI, using a humanized fusion protein atacicept, was initially deemed safe in phase I trials but efficacy has not been determined due to safety concerns during a phase II/III trial which led to its premature termination (44). Modulation of B-cell receptor signaling using epratuzumab (anti-CD22 mAb) has yielded some promising results in non-renal SLE patients although there is currently no data on outcomes in patients with lupus nephritis (45). Recombinant small-molecule inhibitor abatacept, which blocks T-cell co-stimulatory ligands (CD80 and CD86) on B cells and dendritic cells, also failed to meet primary and secondary endpoints in a phase II clinical trial of SLE patients which largely excluded lupus nephritis patients (46).

Despite this lack of success, belimumab, a monoclonal antibody that neutralizes the soluble B-cell activation and survival factor BAFF, has recently been approved for SLE patients with active disease in the USA and Europe by the Federal Drug Administration (FDA) and the European Medicines Agency after successful phase III clinical trials showing modest improvements in disease (47, 48). It is now also available for use in other countries, such as Australia. Belimumab is not only the first targeted therapeutic indicated for SLE but also the first new therapeutic approved for SLE in over 50 years. However, the benefits obtained with belimumab are modest and only attained in patients with mild disease who are already receiving standard therapy (47). Given the overall lack of success of lymphocyte-targeted therapeutics, coupled with the heterogeneity and diverse array of clinical manifestations of disease and the contribution of inflammatory factors to disease development in SLE, more consideration to non-B-cell targets and combination therapeutic regimes are required in order to deliver a more personalized and effective approach to treating SLE. The focus of this review will be to discuss the current understanding of proinflammatory cytokines and myeloid growth and activation factors as well as the promising outcomes and potential for the therapeutic targeting of these factors in SLE.

## Targeting Inflammation in Lupus

### Myeloid-Derived Inflammatory Factors in Lupus Pathogenesis and Targeted Treatment

#### Interleukin-1 and the Inflammasome

The inflammasome is a complex that responds to “danger signals” and induces the cleavage and release of bioactive IL-1, a cytokine upstream of many proinflammatory responses (49). The importance of inflammasome activation and the induction of IL-1 have been elucidated by several studies and have drawn an important link between the innate immune system and autoimmunity (50, 51). Increased *IL-1* gene expression was observed in MRL^lpr/lpr^ mice (52, 53), and inhibition of the NLRP3 inflammasome/IL-1β axis in MRL^lpr/lpr^ mice attenuated proteinuria, autoantibody production, systemic proinflammatory cytokines, and kidney pathology (54, 55). When SLE was induced in mice deficient in IL-1, they showed reduced levels of autoantibody and milder disease manifestations (56). It was also found that IL-1 induced IgG production by cells from SLE patients and healthy controls (57), and increased IL-1 levels were produced by B cells from SLE patients (58) and were found in the cerebrospinal fluid (CSF) of patients with neural SLE (59). A single-nucleotide polymorphism in the *IL1B* gene (rs1143629) is associated with juvenile-onset SLE, implicating IL-1β in the early stages of SLE pathogenesis (60). Treatment of MRL^lpr/lpr^ and NZB/W F_1_ mice with anti-IL-1R antibody reduced autoantibody titers (61, 62). A preliminary study treating patients with the IL-1R antagonist anakinra showed promising results (63); however, no further studies have since been published.

In contrast with the concept of a critical role for inflammasome activation in autoimmunity, inflammasome deficiency due to a point mutation in the *NLRP3* gene has recently been identified in lupus-prone NZB/W F_1_ mice correlating with reduced IL-1β release (64). In addition, the NLRP3/ASC inflammasome protected against kidney damage independent of IL-1 signaling due to regulation of the anti-inflammatory activity of transforming growth factor-β (TGF-β) in C57BL6/J^lpr/lpr^ mice, which represent a mild spontaneous model of lupus (65). Collectively, these studies suggest that therapeutic targeting of the inflammasome in SLE may exacerbate disease in some patients or only be suitable for patients with a certain inflammatory profile. Recently, emphasis has been placed on understanding how the inflammasome contributes to proinflammatory cell death (pyroptosis) and the subsequent modification of autoantigens and generation of autoimmune responses in lupus (66).

#### Interferon Alpha

Interferon alpha (IFN-α) is one of the most strongly implicated cytokines in the pathogenesis of SLE (67). Early studies showed that IFN-α was increased in the serum and found to be associated with increased disease activity (68–71). Correlations were also found between the levels of IFN-α and IC in the serum (69) as well as the deposition of IC in kidney sections from SLE patients (72). This may be due to IFN-α having an antagonistic effect on CRP production, leading to the elevation of available nuclear antigens (23). In addition, IFN-α was expressed at high levels in the CSF of patients with neural lupus (73, 74), and CSF from these patients had strong IFN-α-inducing ability when added to cell cultures (75). Treatment of a patient harboring a malignant tumor with IFN-α induced SLE-like symptoms (76), and it is also epidemiologically interesting that SLE patients with high IFN-α levels showed low rates of hepatitis B infections (77), highlighting the role of IFN-α not only in antitumor and antiviral responses but also in the pathogenesis of SLE. Interestingly, viral DNA and RNA as well as self nucleic acid-containing ICs stimulate IFN-α production by plasmacytoid dendritic cells via TLR-7 and TLR-9; specific inhibitors toward these TLRs potently inhibited IFN-α production (78), and treatment of lupus-prone NZB/W F_1_ mice with these inhibitors significantly reduced serum ANAs, glomerulonephritis, and organ damage while improving survival (79). In addition, increased TLR-7 and TLR-9 expression in PBMCs from SLE patients was correlated strongly with high levels of IFN-α mRNA (80), thus bridging innate immunity with autoimmunity. Clinical assessment of targeting IFN-α has shown safety (81). A phase II study did not reach its primary endpoint; however, exploratory analysis of the results did reveal positive reductions in disease (82). A phase I/II trial in SLE patients examining the safety of IFNα kinoid (IFN-K) that induces a host polyclonal antibody response to IFN-α is currently underway (NCT01058343). A phase I trial in lupus patients testing sifalimumab (MEDI-545), a human anti-IFN-α mAb, showed safety, tolerability, and clinical activity (83), which has led to a phase IIb trial, which has now been completed although no data have been published (NCT00299819). Studies targeting TLRs in nephritic SLE are currently lacking; however, small-molecule TLR antagonists are starting to be assessed for efficacy in animal models (84). Very interestingly, it is now apparent that hydroxychloroquine, a mainstay in lupus treatment, is a TLR-7 and TLR-9 antagonist. Thus, manipulation of TLR signaling with new agents promises to be a future growth area in the clinical management of inflammatory and autoimmune diseases such as SLE [reviewed in Ref. (85)].

#### Interferon Gamma

Interferon gamma (IFN-γ) is a prototypic T_h_1 cytokine that activates a proinflammatory program in macrophages. Like IFN-α, IFN-γ is also elevated in the serum of SLE patients (86–88). Increased IFN-γ levels were also found in lymphoid organs of prediseased MRL^lpr/lpr^ mice (52) while increased IFN-γ-producing T cells were correlated with autoantibody titers and proteinuria in aged diseased mice (89). Treatment of NZB/W F_1_ mice with IFN-γ accelerated disease while neutralization of IFN-γ resulted in reduced disease symptoms and improved survival (90, 91); furthermore, genetic deletion of IFN-γ receptor in these mice impaired autoantibody production and glomerulonephritis (92). Studies in the Lyn^−/−^ mouse model showed that genetic deletion of IFN-γ led to reduced production of BAFF and decreased myeloid proliferation and T-cell hyperactivation, thereby resulting in moderation of glomerular disease (93). Analysis of PBMCs from SLE patients showed that they had significantly higher IFN-γ transcripts compared to control PBMCs (94) and that T cells from SLE patients produced more IFN-γ, which induced BAFF production by monocytes (95), while SLE NK cells produced higher IFN-γ (96). There are currently no completed trials on the effect of neutralizing IFN-γ in SLE patients (97); however, a recent single-dose study treating SLE patients with AMG811, an anti-INF-γ IgG1 mAb, was well tolerated and showed reductions in IFN-γ-mediated gene expression (98).

#### Tumor Necrosis Factor Alpha

Tumor necrosis factor alpha (TNF-α) is an interesting and controversial cytokine in the field of SLE due to its apparent dual role (99). Similar to other proinflammatory cytokines, increased levels of TNF-α have been observed in the serum of lupus-prone animals and SLE patients (100). In particular, elevated serum levels and gene expression levels are positively associated with disease activity as well as renal involvement in SLE patients (88, 101). While TNF-α blockade has been successful as a mainstay treatment for rheumatoid arthritis (102), the assessment of this therapy in SLE patients has not been straightforward. In SLE patient studies, anti-TNF-α therapy increases the serum levels of anti-dsDNA and antiphospholipid autoantibodies (103). It would follow that a further increase in antiphospholipid antibodies in patients may lead to vascular events, which although rare, have the potential to be life-threatening. In addition, the risk of bacterial infection is increased as a result of anti-TNF-α therapy (104). Despite the risks, treatment has led to syndromes that are transient, mild in nature, have not induced flares, and have resulted in reductions in proteinuria and provided benefit to patients with lupus arthritis (105). A more recent study has demonstrated safety and efficacy of anti-TNF-α therapy in SLE (106). It is suggestive that any consideration of anti-TNF-α for the treatment of SLE patients must be for a short duration only and not recommended for patients with antiphospholipid syndrome [up to 15% of SLE patients (107, 108)]. It is still debatable whether the risks associated with therapeutic targeting of TNF-α in SLE are worth the benefits obtained.

#### B-Cell-Activating Factor of the TNF Family

B-cell-activating factor (BAFF) is an important B-cell survival factor with well-known pathogenic roles in SLE. It is expressed by numerous cells in the immune system but is highly expressed by innate immune cells. Mice engineered to overexpress BAFF developed autoimmune manifestations (109) while overexpression of BAFF in lupus-prone congenic strains accelerated renal pathology (110). BAFF levels are increased in the serum of patients with SLE (111, 112) and are associated with increased anti-dsDNA antibody levels (113) and disease activity (114, 115). Neutralization of BAFF in lupus-prone NZB/W F_1_ mice depleted B cells, prevented progressive T-cell activation and dendritic cell accumulation, and prolonged survival (116). Lyn^−/−^ mice show excessive BAFF production by myeloid cells and treatment with anti-BAFF mAb attenuated their lupus-like disease (93). BAFF neutralization also reduced glomerulonephritis and improved survival in lupus-prone BXSB/Yaa mice (117) and NZM2410 mice (118). T cells from SLE patients produced large amounts of BAFF in culture (119) and had significantly upregulated BAFF mRNA (120) compared to control T cells. Increased BAFF expression was also found in SLE B cells and was positively associated with anti-dsDNA autoantibody and disease activity scores (121), indicating that as well as being produced by other cell types, B cells from SLE patients can produce BAFF in an autocrine manner. Especially pertinent is the FDA approval of belimumab (trade name Benlysta^®^), a neutralizing antibody against BAFF, for the treatment of SLE (48), spurred by the success of a randomized double-blind placebo-controlled trial that demonstrated efficacy and safety in SLE patients treated with belimumab over placebo (47). This pathway continues to dominate the focus of SLE clinical trials.

### Myeloid Growth Factors Drive Splenomegaly, Inflammatory Myeloid Phenotypes, and Contribute to the Immunopathology of Lupus Nephritis

Extramedullary hematopoiesis is often a hallmark of infectious and inflammatory diseases, driven by excess production of myeloid growth factors and is evident in models of lupus such as Lyn^−/−^, NZB/W F_1_, and MRL^lpr/lpr^ mice (Figure 1) (15, 122). This phenomenon may contribute to the splenomegaly seen in some lupus patients and lupus-prone mice (15, 122, 123). Myeloid growth factors stimulate progenitor cell release from bone marrow, myeloid cell production, and cellular activation, and this may promote enhanced inflammatory responses and tissue damage depending on tissue context. Several studies have implicated myeloid growth factors, including IL-3, M-CSF, and GM-CSF, in the inflammatory pathways and pathology in SLE.

#### Interleukin-3

Interleukin-3 (IL-3) is a pleiotropic, synergistic growth factor that is involved in the differentiation, activation, and support processes of many immune cells including dendritic cells (124). Although it has long been known that IL-3 can be elevated in SLE patients (125), very few studies on the role of IL-3 in SLE have since been conducted. In the Lyn-deficient mouse model of lupus, IL-3-responsive progenitor cells are elevated in spleen (15), and IL-3 induces enhanced signaling and survival of Lyn^−/−^ plasma cells, suggesting it may play a role in the support of autoreactive plasma cells (126). Recently, a study has shown that IL-3 can drive glomerulonephritis in MRL^lpr/lpr^ mice, hypothesized to be due to enhanced antigen presentation by dendritic cells, elevated Ig secretion, and/or basophil-mediated support functions (127). Treatment of MRL^lpr/lpr^ mice with an anti-IL-3 mAb ameliorated nephritis, improved kidney function, and restrained production of certain autoantibodies (127). This suggests that the IL-3 axis might be an undervalued contributor to inflammation and pathology in SLE, and future studies to further our understanding of this system may benefit the development of superior treatment regimes.

#### Macrophage Colony-Stimulating Factor

Macrophage colony-stimulating factor (M-CSF) is a myeloid growth factor that induces the differentiation of myeloid precursor cells into monocytes/macrophages or dendritic cells as well as regulating macrophage functions, survival, trafficking, and proliferation, and it is associated with inflammatory pathology (128). M-CSF is elevated in the serum of SLE patients and correlates with active disease, renal pathology, and myeloid activation syndrome (129, 130). Local M-CSF production by renal mesangial cells is elevated in lupus nephritis and contributes to proteinuria, local macrophage infiltration and proliferation, and glomerular proliferation (131, 132). M-CSF is also detectable in the urine of lupus nephritis patients with levels correlating with flares in renal disease (133). Elevated M-CSF is also observed in MRL^lpr/lpr^ mice systemically as well as in the kidney (134) and is heavily implicated in driving autoantibody production, glomerular infiltration of myeloid cells and nephritis (135). M-CSF is involved in driving a proinflammatory, immunopathogenic phenotype in MRL^lpr/lpr^ macrophages (136), and in Lyn^−/−^ mice, hematopoietic progenitors responsive to M-CSF are enhanced in spleen (15). Inhibition of M-CSF signaling with a selective M-CSF receptor kinase inhibitor (GW2580) prevents macrophage and T-cell accumulation in the kidney, restricts the local renal inflammatory profile, and improves kidney pathology in an induced model of lupus nephritis (137). This highlights that the M-CSF pathway may be a novel target for therapeutic trials in SLE.

#### Granulocyte–Macrophage Colony-Stimulating Factor

Granulocyte–macrophage colony-stimulating factor (GM-CSF) is a growth factor that drives differentiation of myeloid lineage cells (granulocytes and monocyte/macrophages), and it can act on mature immune cells to upregulate an inflammatory phenotype and enhance antigen presentation and migration (138). Although there is conflicting evidence as to whether GM-CSF levels are altered in SLE (139, 140), the frequency of systemic GM-CSF-secreting immune cells is elevated and correlates with anti-dsDNA titers in SLE (141). It has also been suggested that high concentrations of GM-CSF can drive Ig secretion and leukocyte activation marker CD69 expression in lupus patients (142). GM-CSF can also be produced locally by glomerular mesangial cells and levels correlate with lupus nephritis (131). Interestingly, some studies suggest that neutrophils and dendritic cells may demonstrate resistance to GM-CSF responses in SLE (143, 144). Colony formation by splenic progenitor cells induced by GM-CSF is increased in Lyn^−/−^ mice (15). Therapeutic targeting of the GM-CSF axis is not currently being explored in SLE, but has shown promise in rheumatoid arthritis (138). MOR103, a humanized anti-GM-CSF mAb, has recently successfully completed a phase I/II trial in rheumatoid arthritis reporting safety and preliminary efficacy (145), and a fully human anti-GM-CSF receptor mAb, mavrilimumab, is currently in phase II trial (NCT01712399). Further studies delineating the role of GM-CSF in inflammation and pathology in SLE may highlight this system as a potential target for treatment in a subset of SLE patients.

### Interleukin-6: a Major Mediator of Inflammation in SLE

#### IL-6 Drives Immunopathology in Lupus Nephritis

Interleukin-6 (IL-6) is a pleiotropic cytokine that acts on a range of cell types; it can influence growth and differentiation and antibody production and mediates the acute-phase inflammatory response. It is also highly implicated in the pathogenesis of many inflammatory and autoimmune diseases [reviewed in Ref. (146)]. IL-6 has consistently been shown to be elevated in the serum of SLE patients and it has been suggested that levels correlate with disease activity, making it a suitable biomarker for tracking disease activity (147–150). Similarly, IL-6 can be elevated in the urine of lupus nephritis patients, with higher levels correlating with active renal inflammation and pathology (151, 152). IL-6 has been implicated in driving autoantibody production and loss of tolerance in SLE through the upregulation of recombination-activating gene (RAG) activity (153). Studies have identified polymorphisms in the IL-6 gene, which are associated with SLE susceptibility (154). In mice, elevated levels of IL-6 are observed in numerous models, including NZB/W F_1_ (155), MRL^lpr/lpr^ (156), Lyn^−/−^ (15), ALD-DNA (157), B6.sle1.sle2.sle3 (158), and B6.sle1.Yaa (159), and it is heavily implicated in pathology. Studies in IL-6-deficient mice have shown that they are resistant to ALD-DNA-induced lupus, which ordinarily promotes anti-dsDNA autoantibody titers, proteinuria, CD4^+^ T-cell activation, and glomerulonephritis (157). The mechanism is thought to be due to an expansion of regulatory T cells in the absence of IL-6, which ordinarily suppresses their generation (157). Impaired Treg maturation and activity was observed in B6.sle1.sle2.sle3 mice due to overproduction of IL-6 by dendritic cells (158). IL-6 has also been shown to promote disease in Lyn^−/−^ mice as Lyn^−/−^IL-6^−/−^ show moderated B-cell hyperactivity and plasmacytosis and abrogation of T-cell hyperactivity and splenic myeloid cell expansion (15). In addition, class-switched pathogenic ANAs, glomerular IgG, and complement deposition are absent in Lyn^−/−^IL-6^−/−^ mice, and glomerular structural integrity is significantly improved (15). Similarly, IL-6-deficient MRL^lpr/lpr^ mice showed greatly improved survival with significant amelioration of renal immunopathology (160), and in B6.Sle1.Yaa mice, IL-6 deficiency eliminated autoantibody production and nephritis (161).

**Figure 3:**
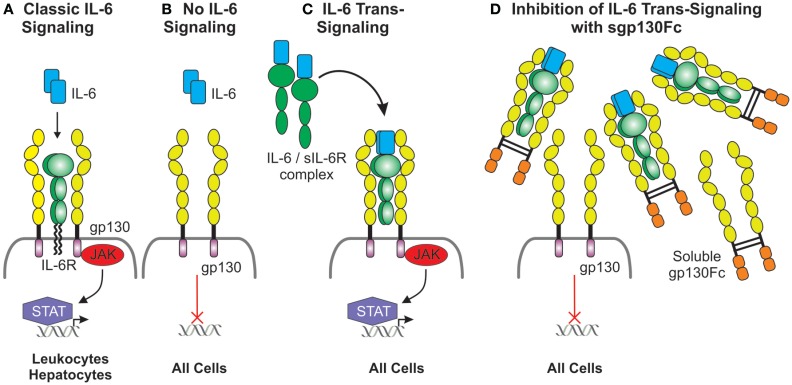
**Duplicitous signaling roles of IL-6**. **(A)** Classical IL-6 signaling occurs via direct interaction of IL-6 with the membrane-bound IL-6 receptor, which has a limited cellular distribution, and the ubiquitously expressed gp130. gp130 lacks intrinsic kinase activity; IL-6 signals are transduced intracellularly via the recruitment and activation of the JAK/STAT pathway. **(B)** Cells lacking the IL-6 receptor are not receptive to IL-6 except **(C)** in the presence of the IL-6/soluble IL-6 receptor complex which interacts with gp130 expressing cells and this is defined as IL-6 trans-signaling. **(D)** Inhibition of IL-6 trans-signaling can be achieved via the presence of excess soluble gp130Fc fusion protein.

It has been hypothesized that classic IL-6 signaling via membrane-bound IL-6R (Figures 3A,B) does not contribute to chronic inflammation but mediates regulated pathogen clearing processes through anti-inflammatory pathways. However, IL-6 trans-signaling through soluble IL-6R/gp130 complexes (Figure 3C) is believed to be associated with chronic inflammatory conditions by driving hyperinflammatory and immunopathogenic processes (162). Specific downregulation of IL-6 trans-signaling in Lyn^−/−^ mice through transgenic overexpression of soluble gp130-Fc fusion protein (Lyn^−/−^sgp130FcTg) resulted in a loss of splenomegaly, a decrease in splenic myeloid cells, and reduced systemic BAFF levels (163). Although pathogenic ANA production was sustained, as was IgG IC deposition in kidney glomeruli, renal complement deposition was significantly reduced, which suppressed renal leukocyte infiltration, thereby markedly attenuating glomerulonephritis and improving kidney function (163). These findings suggest that more emphasis should be put on examining the role of IL-6-trans-signaling in lupus nephritis, with the possibility of targeting this inflammatory pathway in disease.

#### Targeting IL-6 has Therapeutic Potential

There is much evidence to suggest that IL-6 plays a significant role in a number of inflammatory, autoimmune, and proliferative diseases, making it a strong candidate for targeted novel biological therapeutics [reviewed in Ref. (164)]. This notion is further enhanced by the improvement seen in IL-6-targeted murine SLE models. A preliminary study treating NZB/NZW F_1_ mice monthly with anti-IL-6 in combination with anti-IL-1α concluded that this regime only had a partial effect on disease, alleviating proteinuria (165). Young NZB/NZW F_1_ mice treated weekly with a rat anti-IL-6 monoclonal antibody from 3 to 9 months of age showed reduced ANA production, improved proteinuria, and increased survival, although these mice required initial tolerizing treatments of anti-CD4 to prevent a rapid-onset anti-rat Ig response (166). A more thorough study in young NZB/W F_1_ mice without anti-CD4 tolerance showed that anti-IL-6 mAb treatment suppressed systemic serum amyloid A (SAA) and anti-dsDNA antibody levels, suppressed hyperactivation of B and T cells, and greatly diminished the development of kidney pathology (167).

The use of murine IL-6 targeting monoclonal antibodies in human disease has been unsuccessful as IL-6/mAb immune complexes form which can further drive pathology in inflammatory settings (168). A fully humanized anti-IL6 mAb, sirukumab, has shown tolerance in humans (169, 170) and efficacy in treating rheumatoid arthritis (171). A phase I trial in cutaneous and systemic lupus patients (NCT01702740) concluded that sirukumab is generally well tolerated in SLE although mild leukopenia, neutropenia, and decreases in platelet count were observed (172). A phase II trial in lupus nephritis patients (NCT01273389) has recently been completed with official outcomes not yet reported, although unofficial accounts indicate a high frequency of serious adverse events, the majority of which were infections.

A study examined the effect of targeting IL-6R in MRL^lpr/lpr^ mice by treating 15-week-old animals every 1–3 days for 5 weeks with a neutralizing rat anti-IL-6R mAb. These mice saw an initial reduction in anti-dsDNA autoantibodies, which rebounded and elevated in response to increasing titers of anti-rat Ig antibodies (173). The treatment was reported as successful as both kidney function and structure was improved at the endpoint compared to controls (173). Tocilizumab, a humanized murine anti-IL6-R mAb, has demonstrated efficacy in treating multiple myeloma (174) and rheumatoid arthritis (175). A phase I safety trial demonstrated that tocilizumab is well tolerated in SLE patients although similarly to sirukumab, temporary dose-dependent neutropenia was observed (176). A recent study in SLE patients showed that tocilizumab treatment can reduce B- and T-cell activation, memory B cells, and autoantibody-producing plasma cells without impacting naive B cells populations (177).

Given that anti-IL-6 and anti-IL6R inhibition may lead to unwanted neutropenia and infection, targeting IL-6 trans-­signaling without disrupting classical signaling pathways may prove to be a superior approach in SLE as many anti-inflammatory and regulatory mechanisms mediated by IL-6 classic signaling will not be impacted (Figure 3D) (162, 163, 178). A fusion protein joining sgp130 to the IgG Fc region, sgp130Fc, has demonstrated effective inhibition of soluble IL-6R-mediated trans-signaling (178). Proof of concept for sgp130Fc treatment has been demonstrated in a range of inflammatory disease models, including rheumatoid arthritis (179, 180), atherosclerosis (181), and Crohn’s disease (178). Given its efficacy, it is surprising that no clinical trials utilizing this agent in lupus appear to be in the pipeline.

Components of signaling pathways that are aberrantly activated in disease are attractive drug targets. The JAK/STAT signaling pathway is activated downstream of IL-6 and gp130 (Figures 3A,C) and can be targeted by small-molecule inhibitors, which have already shown promise in cancer therapy (182). However, there are few studies on JAK/STAT inhibitor treatments in inflammatory diseases; one study demonstrated clinical efficacy of the JAK1/2 inhibitor AZD1480 in five models of experimental autoimmune encephalitis (EAE, modeling human multiple sclerosis) by inhibiting myeloid cell hyperactivation, T_h_1 and T_h_17 differentiation, and proinflammatory cytokine production as well as showing improvements in disease and clinical pathology (183). A signaling study in Lyn^−/−^ mice showed that treatment with AZD1480 resulted in reduced splenic B cells and plasma cells as well as reduced numbers of splenocytes and thymocytes; however, effects on lupus pathology were not reported (126). Although this may be an attractive approach, inhibition of the JAK/STAT pathway in autoimmune disease should be undertaken cautiously and the use of single-specificity inhibitors may be more beneficial. Indeed, treatment using the JAK2-selective inhibitor CEP-33779 in both NZB/W F_1_ and MRL^lpr/lpr^ mice saw dose-dependent improvements in lymphadenopathy and splenomegaly, reductions in systemic C3 and proinflammatory cytokines, including IL-1, IL-12, IFN-α, IL-17A, and TNF-α, reductions in autoantibody-producing plasma cells, increased survival, and significant improvements in glomerulonephritis through blockade of STAT3-mediated signaling (184, 185). These animal data lend support for trailing a JAK2 inhibitor in human disease. Interestingly, GSK recently halted development of the JAK1 inhibitor GSK2586184 following disappointing results in a phase II study in SLE (186).

## Future Directions

### Targeting Multiple Aberrant Pathways with Combination Therapies

Although the pathogenic pathways involved in SLE are still being defined, it is evident that a combination of deregulated processes are necessary for the disease to manifest. As pathogenesis in SLE is clearly multifactorial, the targeting of a single pathway or factor may not sufficiently alter the progression of disease. Indeed, current treatment utilizes both agents that suppress the immune response alongside those that dampen inflammation (38). One of the reasons that B-cell-targeted therapies have been largely unsuccessful in SLE trials is that they fail to target pathogenic long-lived plasma cells and memory B cells (187). Recent studies in NZB/W F_1_ mice treated with a B cell targeting anti-CD20 mAb alongside a plasma cell-depleting agent (188) or BAFF-blocking agent (189) resulted in superior improvements in disease compared to B-cell depletion on its own. Similarly, depletion of memory B cells using an anti-IL-15 mAb alongside TACI-Ig, a soluble form of the TACI receptor that binds to BAFF and APRIL ligands, was more efficacious than TACI-Ig alone (190). This illustrates that combining targeted therapeutics can give rise to better outcomes. As systemic inflammation is essential for disease propagation and sustenance, controlling both the proinflammatory arm alongside pathogenic plasma or memory B-cell depletion may prove to be a superior therapeutic approach to disease control in SLE.

## Conclusion

Systemic lupus erythematosus is a highly complex autoimmune disease with multiple immunopathological disorders, the most significant being glomerulonephritis mediated by immune complex deposition in the kidney microvessels. Although autoreactive B cells are essential, disease progression relies on the establishment of a systemic inflammatory environment largely generated by myeloid cells and T lymphocytes. Current treatment utilizes broad-spectrum immunosuppressive and anti-inflammatory agents which have many off-target effects. Therapeutic targeting of the B-cell compartment in SLE has only had limited success, and a need for novel targeted therapeutics is essential for personalized, effective control of disease to improve quality of life. Many therapeutics targeting inflammatory cytokines, growth factors, and signaling pathways have shown safety and efficacy in inflammatory disorders, and preliminary studies in animal models of murine lupus and human lupus nephritis patients have demonstrated some promising disease-suppressing effects. Presently, there are no therapies for human SLE in clinical practice that target specific inflammatory pathways and thus further studies elucidating the mediators and their mode of action in generating the pathogenic inflammatory environment in lupus will greatly benefit the selection and development of therapeutics for future clinical trials.

## Conflict of Interest Statement

The authors declare that the research was conducted in the absence of any commercial or financial relationships that could be construed as a potential conflict of interest. The Guest Associate Editor, Dr. Harris, declares that, despite being affiliated with the same institution as the authors, the review process was handled objectively.

## Abbreviations

ANAs, antinuclear antibodies; APPs, acute-phase proteins; auto-Abs, autoantibodies; BAFF, B-cell-activating factor of the TNF family; CRP, C-reactive protein; IC, immune complex; IFN, interferon; Ig, immunoglobulin; IL, interleukin; mAb, monoclonal antibody; SLE, systemic lupus erythematosus/lupus; T_h_, T helper; TLR, toll-like receptor; T_reg_, regulatory T cell.
